# Ruptured abdominal aortic aneurysm discovered by pocket-sized ultrasound in a low resource setting: a case report

**DOI:** 10.1186/s12245-023-00579-w

**Published:** 2024-01-17

**Authors:** Ihab Alasasfeh, Rawan Abudawood, Bayan E.Hwidi, Raghad Al-Shami

**Affiliations:** 1 Department of General Surgery, Emergency Medicine Unit/Jordan University Hospital, School of Medicine, The University of Jordan, Amman, Jordan; 2 School of Medicine, The University of Jordan, Amman, Jordan

**Keywords:** POCUS, Point-of-care ultrasound, Pocket-sized ultrasound, Ruptured abdominal aortic aneurism

## Abstract

**Background:**

Abdominal aortic aneurysm (AAA) is a life-threatening condition characterized by the weakening and dilation of the abdominal aorta. AAA primarily affects men, smokers, and the elderly, with rupture being a fatal complication. While point-of-care ultrasound (POCUS) is valuable in diagnosing AAA, the role of using pocket-sized ultrasound in a low resource setting remains less explored. This case report presents a unique instance of a suspected ruptured AAA diagnosed using pocket-sized ultrasound in an emergency department (ED) situated in a low resource setting where ultrasound machines are absent, and emergency physicians lack proficiency in ultrasound usage.

**Case presentation:**

A 78-year-old man with a history of hypertension and bladder cancer presented to the ED with suprapubic pain. Initial evaluation showed no concerning findings. However, the next day, he collapsed, became unconscious, and experienced a cardiac arrest. Despite resuscitation efforts, the patient’s condition deteriorated. POCUS revealed an 8-cm dilated abdominal aorta with an intimal flap, indicative of a dissecting AAA and a substrate for AAA rupture. Unfortunately, the patient died despite resuscitation efforts.

**Conclusion:**

This case highlights the importance of considering AAA in patients with risk factors and abdominal pain in a low resource setting. POCUS using a pocket-sized ultrasound can aid in early AAA detection, potentially preventing rupture through preemptive vascular intervention. Emergency departments should prioritize ultrasound availability, and emergency physicians should be proficient in its use.

## Background

Abdominal aortic aneurysm (AAA) is a potentially life-threatening condition characterized by weakening and dilation of more than 3 cm of the abdominal aorta [1, 2]. AAA affects men and smokers more commonly and its prevalence increases with age [3, 4]. While AAA can develop asymptomatically, if ruptured, it can manifest as sudden and severe pain in the abdomen, back, or flank, often requiring urgent medical intervention. Delay in diagnosis and treatment contribute to the high mortality of the disease with rupture being the most fatal complication [5–7]. In acute settings of suspected AAA, POCUS can be used with high accuracy [8]. However, less is known about the use of pocket-sized ultrasound to diagnose AAA in a low resource setting. We report a case of a suspected ruptured AAA identified by a pocket-sized ultrasound in the emergency department (ED) in a low resource setting lacking an ultrasound machine. Furthermore, the Butterfly ultrasound device used in the diagnosis is a personal device for the attending physician on that day, highlighting the limited availability of such equipment. Moreover, the absence of training programs for emergency doctors in ultrasound usage is a challenge we face. Additionally, it is worth mentioning that there is only one computerized tomography (CT) machine for clinic patients, inpatients, and emergency patients, which frequently malfunctions due to workload pressure. Besides, there is a shortage of staff members. For example, each nurse is assigned to 5 beds in the ED, and the vascular surgeon consulted was a part-timer, indicating limited full-time availability.

## Case presentation

A 78-year-old man presented to the ED complaining of suprapubic pain that started early in the morning and became progressively worse, pain was not radiating anywhere, and was associated with frequency, with no other lower urinary tract symptoms, fever, or chills. Triage assessment was level 4 semi-urgent. His medical history was remarkable for hypertension, benign prostatic hyperplasia, and a history of low-grade bladder ca diagnosed in 2013.

His Glasgow Coma Scale (GCS) was 15, Blood pressure was 124/60, pulse was 77/min, respiratory rate was 20/min, oxygen saturation was 98% on room air, and temperature was 37 °C. The patient was conscious, alert, and oriented to place, time, and person. His abdominal examination revealed a soft lax abdomen without tenderness and no costovertebral angle tenderness. Complete blood count, kidney function test, urine analysis, and urine culture were done, and all were normal, except for anemia with a hemoglobin level of 12.9 g/dL and potassium level near the lower normal level with a value of 3.34 mmol/L. The patient was given IV Paracetamol, IV Hyoscine butyl bromide, IM diclofenac, and normal saline. He was then referred to a urology clinic and discharged.

On the next day, the patient had severe loin pain in the morning, then suddenly collapsed and lost consciousness as reported by his family. He was then transferred to the ED by ambulance. In the ED the patient was unconscious with a GCS of 6/15 (E4 V1 M1), both blood pressure and O2 saturation were not recordable, pulse was intact, and pupils were both mid-dilated with sluggish reactivity to light. Intubation with rapid sequence intubation was performed. Post-intubation electrocardiogram demonstrated normal sinus rhythm without acute ST changes. The patient subsequently became pulseless, 1 cycle of CPR was done with 1 mg of Epinephrine, sodium bicarbonate, and calcium gluconate were given. After that, the patient reverted and was started on noradrenaline at a maximum dose with blood pressure recorded at 80/50. Labs were sent for type and cross for blood transfusion, complete blood count, C-reactive protein, kidney function test, electrolytes, troponin, and BNP.

**Fig. 1:**
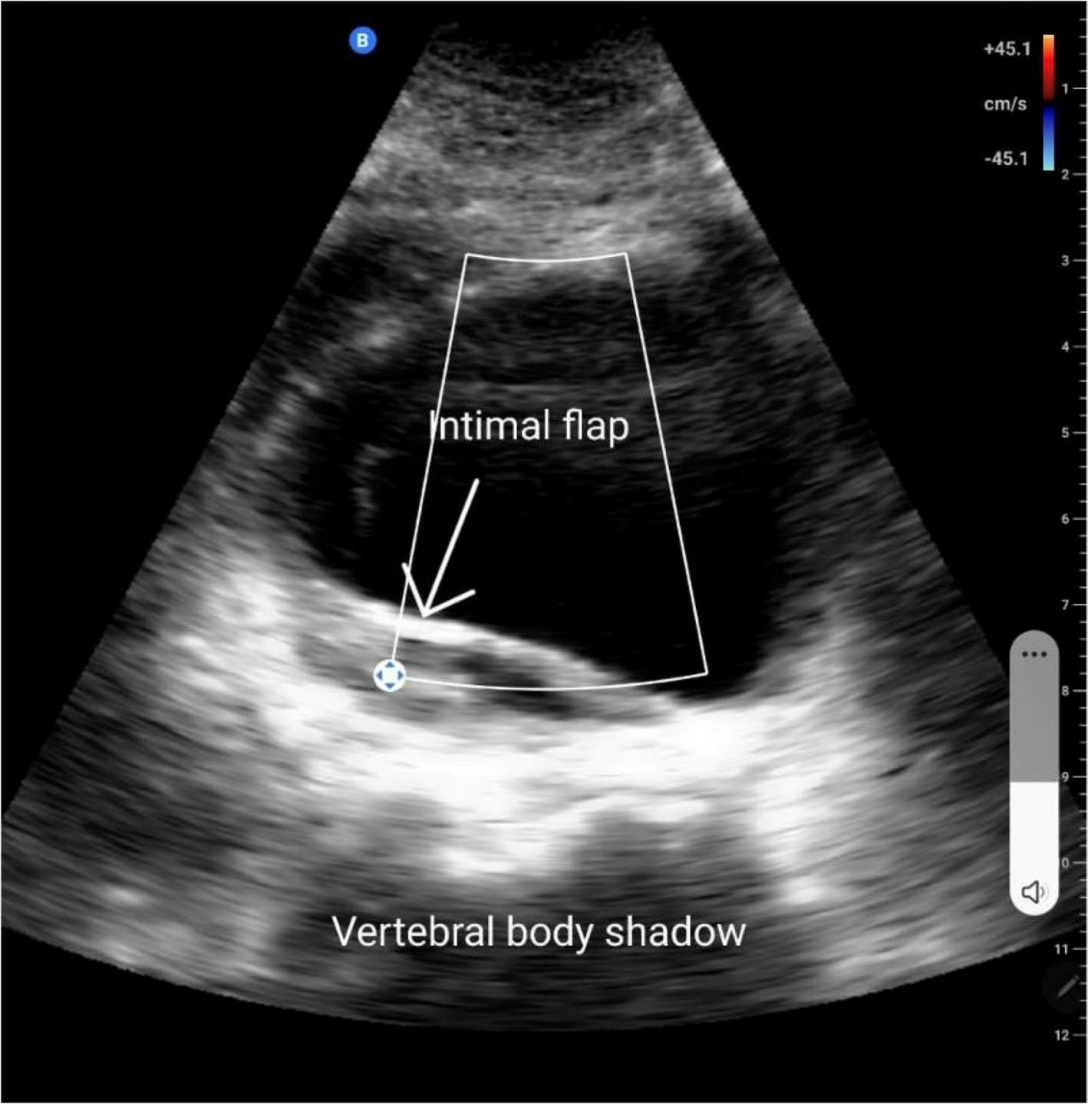
A screenshot taken from the tablet connecting to the butterfly IQ probe showing about 5 cm aortic aneurism at the level of mid-aorta taken in transverse view with a large intimal flap

Four minutes later, he was arrested again, another 1 CPR cycle with adrenaline was done and the patient reverted again. Post-CPR electrocardiogram again demonstrated normal sinus rhythm without acute ST changes and the results of venous blood gas on the ventilator were PH = 7.295/pCO2 = 23.9 mmHg/cHCO3 = 11.4 mEq/L/ctCO2 = 12.1 mmol/L/BE (base excess) =  − 13/pO2 = 138 mmHg/SO2 = 98%. During that, we noticed that his lower limbs were paler and colder than his upper limbs, thus carotid and femoral pulses were examined, and the examination showed a diminished femoral pulse when compared to the carotid pulse. Point of care ultrasound was done using the “Butterfly IQ ultrasound probe,” which is a portable pocket-sized probe that can be easily connected to a phone or a tablet (image shown in Fig. 1), and found a dilated abdominal aorta, around 8 cm, with an intimal flap. Despite starting a massive blood transfusion and giving 1 g of tranexamic acid, the vascular surgery team was not available until about one hour later, while they evaluated the patient in ED, his condition was extremely unstable. During that, laboratory results were out, and hemoglobin level dropped from 12.9 g/dL to 10.2 g/dL within a day, PO_4_ = 5 mg/dl, troponin = 64 ng/L. The patient was arrested again, and 8 cycles of CPR were done with adrenaline 1 mg given 4 times, the patient had pulseless electrical activity during the whole resuscitation process. Unfortunately, the patient died despite all these efforts.

## Discussion and conclusion

Abdominal aortic aneurysm is the focal dilation of the abdominal aorta of more than 3 cm in ultrasound or CT imaging [2]. It most commonly occurs among patients above the age of 65. Moreover, a meta-analysis conducted in 2013 showed that its prevalence is higher in men compared to women [9]. One of the risk factors includes smoking; being the most important risk factor for AAA with a prevalence of 1 in 9 for current smokers compared to 1 in 17 in the general population [4]. Male sex, advanced age, atherosclerosis, hypertension, hypercholesterolemia, positive family history, and trauma are all risk factors for the disease [10]. The diameter of the abdominal aorta is the best predictor factor for the risk of rupture, being almost nonexistent when the diameter is less than 4. However, the risk increases with the increase of the diameter, reaching 30–50% when it reaches 8 cm [11].

Ruptured abdominal aortic aneurysm is considered a vascular surgical emergency, in which unfortunately 50% of patients die even before reaching the hospital, with an overall mortality rate reaching up to 80–90% [12]. Most AAAs are asymptomatic until they rupture [13]. This highlights the importance of screening those with risk factors. The USPSTF (United States Preventive Services Task Force) recommends 1-time screening for abdominal aortic aneurysm (AAA) with ultrasonography in men aged 65 to 75 years who have never smoked. An abdominal aortic aneurysm can be diagnosed using ultrasound. A systemic review showed that the Sensitivity and specificity of the ED US for the detection of AAA were reported to be 99% and 98% respectively [14]. Ruptured AAA is the likely diagnosis in the setting of shock and sonographic identification of AAA However, ED US is much less useful in identifying rupture since 88% of AAAs rupture into the retroperitoneal space [15]. Thus, Computed tomography with contrast is still considered the diagnostic modality of choice for ruptured AAA. However, because it is less available, particularly in a low resource setting, is time-consuming, and usually needs the patient to be transferred out of the ED, the use and importance of (POCUS) has increased more and more.

In conclusion, our case highlights the importance of excluding AAA in those with risk factors complaining of any abdominal pain, this can be easily done using a hand-held Ultrasound probe. Unfortunately, in the case reported here, the patient’s AAA remained undiagnosed until he presented to the ED with suspected ruptured AAA despite his presentation the day before complaining of suprapubic pain. A screening ultrasound in his first presentation even with low-cost probes, would likely have identified his AAA before rupture and allowed him to be evaluated for surgical repair. But, due to the absence of such devices in our Emergency Department and the lack of professional ultrasound skills among emergency physicians in addition to the unavailability of immediate intervention caused by the lack of a full-time vascular surgeon unfortunately the patient was lost. In conclusion, ultrasound must be available in all emergency departments, or at the minimal settings with ultra-portable machines with mobile or tablet, and all emergency physicians must inquire about the skills needed to use it proficiently.

## Data Availability

All data generated or analyzed during this study are included in this published article.

## Abbreviations

AAA: Abdominal aortic aneurysm
RSI: Rapid sequence intubation
US: Ultrasound
POCUS: Point-of-care ultrasonography
GCS: Glasgow Coma Scale
USPSTF: United States Preventive Services Task Force

## References

[1] Johnston KW, Rutherford RB, Tilson MD, Shah DM, Hollier L, Stanley JC (1991). Suggested standards for reporting on arterial aneurysms. Subcommittee on Reporting Standards for Arterial Aneurysms, Ad Hoc Committee on Reporting Standards, Society for Vascular Surgery and North American Chapter, International Society for Cardiovascular Surgery. J Vasc Surg.

[2] Chaikof EL, Dalman RL, Eskandari MK, Jackson BM, Lee WA, Mansour MA, Mastracci TM, Mell M, Murad MH, Nguyen LL, Oderich GS, Patel MS, Schermerhorn ML, Starnes BW (2018). The society for vascular surgery practice guidelines on the care of patients with an abdominal aortic aneurysm. J Vasc Surg.

[3] Haque K, Bhargava P (2022). Abdominal aortic aneurysm. Am Fam Physician.

[4] Tang W, Yao L, Roetker NS, Alonso A, Lutsey PL, Steenson CC, Lederle FA, Hunter DW, Bengtson LG, Guan W, Missov E, Folsom AR (2016). Lifetime risk and risk factors for abdominal aortic aneurysm in a 24-year prospective study: the ARIC study (Atherosclerosis Risk in Communities). Arterioscler Thromb Vasc Biol.

[5] Stather PW, Sidloff DA, Rhema IA, Choke E, Bown MJ, Sayers RD (2014). A review of current reporting of abdominal aortic aneurysm mortality and prevalence in the literature. Eur J Vasc Endovasc Surg.

[6] Sidloff D, Stather P, Dattani N, Bown M, Thompson J, Sayers R, Choke E (2014). Aneurysm global epidemiology study: public health measures can further reduce abdominal aortic aneurysm mortality. Circulation.

[7] Sebesta P, Klika T, Zdrahal P, Kramar J (1998). Ruptured abdominal aortic aneurysm: role of initial delay on survival. J Mal Vasc.

[8] American College of Emergency Physicians (2006). Emergency ultrasound imaging criteria compendium. American College of Emergency Physicians. Ann Emerg Med.

[9] Li X, Zhao G, Zhang J, Duan Z, Xin S (2013). Prevalence and trends of the abdominal aortic aneurysms epidemic in general population–a meta-analysis. PLoS ONE.

[10] Owens DK, Davidson KW, Krist AH, Barry MJ, Cabana M, Caughey AB, Doubeni CA, Epling JW, Kubik M, Landefeld CS, Mangione CM, Pbert L, Silverstein M, Simon MA, Tseng CW, Wong JB, US Preventive Services Task Force (2019). Screening for abdominal aortic aneurysm: US preventive services task force recommendation statement. JAMA.

[11] Brewster DC, Cronenwett JL, Hallett JW, Johnston KW, Krupski WC, Matsumura JS, Joint Council of the American Association for Vascular Surgery and Society for Vascular Surgery (2003). Guidelines for the treatment of abdominal aortic aneurysms. Report of a subcommittee of the joint council of the american association for vascular surgery and society for vascular surgery. J Vasc Surg.

[12] Robinson WP, Schanzer A, Li Y, Goodney PP, Nolan BW, Eslami MH, Cronenwett JL, Messina LM (2013). Derivation and validation of a practical risk score for prediction of mortality after open repair of ruptured abdominal aortic aneurysms in a US regional cohort and comparison to existing scoring systems. J Vasc Surg.

[13] Aggarwal S, Qamar A, Sharma V, Sharma A (2011). Abdominal aortic aneurysm: a comprehensive review. Exp Clin Cardiol.

[14] Rubano E, Mehta N, Caputo W, Paladino L, Sinert R (2013). Systematic review: emergency department bedside ultrasonography for diagnosing suspected abdominal aortic aneurysm. Acad Emerg Med.

[15] Lech C, Swaminathan A (2017). Abdominal aortic emergencies. Emerg Med Clin North Am.

